# The Association Between Bariatric Surgery and Surgical Outcomes Following Open Carpal Tunnel Release

**DOI:** 10.7759/cureus.80350

**Published:** 2025-03-10

**Authors:** Nicholas B Pohl, Ryan Garemani, Evan Derector, Rick Tosti, Pedro K Beredjiklian, Daniel J Fletcher

**Affiliations:** 1 Division of Hand Surgery, Rothman Orthopaedic Institute, Philadelphia, USA; 2 Division of Hand Surgery, Thomas Jefferson University Hospital, Philadelphia, USA

**Keywords:** bariatric surgery, carpal tunnel release, obesity, surgical outcomes, carpal tunnel syndrome

## Abstract

Introduction: While previous research has evaluated surgical outcomes following open carpal tunnel release (CTR) in obese patients, there is relatively minimal literature regarding outcomes in patients who have previously undergone bariatric surgery prior to open CTR. The purpose of this study is to compare the postoperative functional and surgical outcomes in patients who undergo open CTR with or without a history of bariatric surgery.

Methods: Adult patients with a documented history of bariatric surgery undergoing open CTR surgery between 2015 and 2022 were propensity matched with control open CTR patients with no bariatric surgery history. Patients were matched based on age, sex, race, body mass index (BMI), Charlson Comorbidity Index (CCI), smoking status, and history of diabetes mellitus. A retrospective chart review was performed to collect demographic data, preoperative nerve conduction studies (NCS), surgical characteristics, complications, further treatment, and patient-reported outcome measures (PROMs).

Results: A total of 42 patients having undergone bariatric surgery prior to CTR and 84 control CTR patients with no history of bariatric surgery were included. More bariatric surgery patients demonstrated mild carpal tunnel syndrome (CTS) on preoperative NCS. The overall minor complication rate was similar between patients with and without bariatric surgery history. There were no differences in postoperative Physical Component score (PCS-12) and Mental Component score (MCS-12) as well as no change in PCS-12 scores (ΔPCS-12) between preoperative and one-year postoperative scores.

Conclusions: Patients with a history of bariatric surgery tended to have a larger benefit from open CTR in comparison to the control cohort; however, this was not statistically significant. The current literature regarding CTR outcomes as it relates to obesity and bariatric surgery is limited, and therefore, the association should be explored further with larger patient cohorts.

## Introduction

Carpal tunnel syndrome (CTS) is the most common upper extremity neuropathy which is estimated to occur in 3.8% of the general population [1,2]. When severe, or if nonoperative treatment fails, CTS may be treated through open or endoscopic carpal tunnel release (CTR), found to provide positive outcomes in 75% of patients postoperatively [3]. Obesity has been linked to the development of CTS due to various pathophysiological mechanisms [4]. Increased weight may lead to enhanced mechanical compression of the median nerve due to adipose tissue and vascular factors. Metabolic syndrome, which is associated with obesity, causes endothelial dysfunction, abnormal tissue repair, and modifications in the microcirculation independent of mechanical compression [4].

Obesity is currently a global health concern with two in five adults estimated to be overweight or obese [5]. Additionally, obesity is associated with other medical conditions such as type 2 diabetes, cardiovascular disease, and osteoarthritis [6,7]. While medical or multimodal options have become more prevalent in the management of obesity, bariatric surgery has been an available treatment for the past 50 years [8,9]. Previous research has shown bariatric surgery to not only be an effective method for weight loss but also improve glycemic control in diabetic patients and improve outcomes in orthopedic procedures such as total knee or hip arthroplasty [10,11]. In 2021 alone, the American Society for Metabolic and Bariatric Surgery estimated that 262,893 bariatric surgery procedures were performed in North America, marking a substantial 60% increase since 2011 [12]. However, 5-16% of patients who undergo bariatric surgery have been shown to experience a postoperative neurologic complication, with the development of peripheral neuropathies being the most common form [13].

Despite the increase in obese individuals seeking bariatric surgery and its known association with developing peripheral neuropathy postoperatively, there remains a scarcity of literature on the postoperative outcomes of CTR in patients who have undergone bariatric surgery. The purpose of this study is to compare the postoperative functional and surgical outcomes (patient-reported outcome measures (PROMs), complications, and further treatment) in patients who undergo open CTR with or without a history of bariatric surgery. As the incidence of obesity and bariatric surgery continues to increase, this study may provide patients and hand surgeons with better knowledge pertaining to CTR outcomes following bariatric surgery.

## Materials and methods

After obtaining approval from the Thomas Jefferson University Institutional Review Board (approval number: 2023-2799), this retrospective study was performed at Thomas Jefferson University Hospital located in Philadelphia, Pennsylvania, United States. Adult patients with a documented history of bariatric surgery undergoing open CTR surgery between 2015 and 2022 were included in this study. A Structured Language Query (SQL) was performed using the Common Procedure Code (CPT) 64721 to identify patients who underwent open CTR. Additional keyword searches in the SQL relating to bariatric surgery were performed to further identify patients with documented bariatric surgical history. Bariatric surgical history was confirmed through a manual chart review of the electronic medical record (EMR). Patients undergoing open CTR and with no prior history of bariatric surgery were also identified in order to perform a propensity score match of CTR patients with a history of bariatric surgery. Patients under 18 years of age, with incomplete medical records with missing data, who did not undergo an open CTR, or who underwent revision CTR or additional concurrent procedures at the time of CTR were excluded. 

Once patients were identified, information was collected through additional chart review regarding demographics, preoperative nerve conduction studies (NCS), surgical characteristics, complications, further treatment, and PROMs. Complications, further treatment, and PROMs were evaluated at one year postoperatively. The complications evaluated included infection, persistent symptoms, recurrent symptoms, and other unspecified complications. Demographic and medical history data included age, sex, body mass index (BMI), race, Charlson Comorbidity Index (CCI), smoking status, and history of diabetes mellitus. In terms of NCS grading severity, the designation of mild, moderate, and severe grading was assigned to patients as noted in surgeon preoperative documentation or NCS report findings.

All patients completed perioperative self-reported health outcomes, which included the Medical Outcomes Study 12-Item Short-Form Survey (SF-12). The components of the SF-12 analyzed were the Physical Component score (PCS-12) and the Mental Component score (MCS-12). Both components are scored from 0 to 100, with a higher score indicating better function. The scores are structured to a national norm with a scoring mean of 50 and a standard deviation of 10 [14].

A 2:1 propensity match was performed to match patients with a history of bariatric surgery undergoing open CTR with control open CTR patients with no bariatric surgery history. Only a 2:1 match was performed due to the limitations of the variables included in the matching process. The cohorts were matched based on age, sex, race, BMI, CCI, smoking status, and history of diabetes mellitus. Statistical analysis was performed using t-tests or Mann-Whitney U tests to compare continuous data and chi-squared or Fisher's exact tests to compare categorical data. All statistical analyses were completed using RStudio (Version 4.1.2, R Foundation for Statistical Computing, Vienna, Austria (https://www.R-project.org/)), and a p-value of <0.05 was considered statistically significant. To ensure no potential bias in performing this study, all potential conflicts of interest were disclosed. Although the two authors have financial relationships with medical companies, this study did not receive funding from these companies; additionally, the subject matter was not related to any products developed by these companies. Aspects of the study including data handling and result validation were independently reviewed by co-authors with no industry affiliations to remove any further potential bias.

## Results

In total, 126 patients were included in the final cohort for analysis, with 42 patients having undergone bariatric surgery prior to open CTR and 84 control CTR patients with no history of bariatric surgery. Of the patients with a history of bariatric surgery, 27 (64.3%) patients underwent a gastric bypass procedure, 12 (28.6%) underwent gastric sleeve, two (4.8%) underwent lap band placement, and one (2.4%) was unspecified in the medical record. Propensity match results allowed for no differences between the final cohorts in terms of age (p=0.636), sex (p=0.623), race (p=0.976), BMI (p=0.957), CCI (p=0.752), smoking status (p=0.940), and history of diabetes mellitus (p=0.834) (Table 1). 

**Table 1:** Demographics and NCS results *Indicates statistical significance at p<0.05. Data is presented as mean (standard deviation) and N (%). 2:1 propensity match of patients with and without a history of bariatric surgery was performed based on age, sex, race, BMI, CCI, smoking status, and history of diabetes mellitus. BMI: body mass index; CCI: Charlson Comorbidity Index; NCS: nerve conduction study; EMG: electromyography; CTS: carpal tunnel syndrome; CuTS: cubital tunnel syndrome

Variable	CTR only (N=84)	Bariatric surgery and CTR (N=42)	P-value
Age	57 (14.1)	56.6 (10.8)	0.636
Sex			
Male	25 (29.8%)	10 (23.8%)	0.623
Female	59 (70.2%)	32 (76.2%)	
Race			
White	52 (61.9%)	25 (59.5%)	0.976
Black	11 (13.1%)	6 (14.3%)	
Other	10 (11.9%)	6 (14.3%)	
Unknown	11 (13.1%)	5 (11.9%)	
BMI	35.3 (7.03)	36.2 (9.11)	0.957
CCI	2.27 (1.84)	2.24 (1.92)	0.792
Smoking			
Non-smoker	59 (70.2%)	31 (73.8%)	0.940
Former	21 (25%)	9 (21.4%)	
Current	4 (4.76%)	2 (4.76%)	
Diabetes mellitus	25 (29.8%)	11 (26.2%)	0.834
Laterality			
Right	57 (67.9%)	24 (57.1%)	0.324
Left	27 (32.1%)	18 (42.9%)	
Follow-up length (months)	1.33 (1.78)	1.33 (2.38)	0.378
Preoperative NCS (EMG/NCS)	77 (91.7%)	40 (95.2%)	0.717
NCS: CTS results			
CTS mild	0 (0%)	4 (10%)	0.029*
CTS moderate	35 (45.5%)	18 (45%)	
CTS severe	35 (45.5%)	17 (42.5%)	
Unknown	7 (9.09%)	1 (2.5%)	
NCS: CuTS results			
CuTS mild	0 (0%)	1 (2.5%)	0.342
NCS: ulnar nerve at wrist results			
Ulnar nerve wrist mild	1 (1.3%)	0 (0%)	1.000
Ulnar nerve wrist moderate	1 (1.3%)	0 (0%)	
Ulnar nerve wrist severe	1 (1.3%)	0 (0%)	
Contralateral NCS: CTS results			
CTS mild	2 (2.6%)	6 (15%)	0.036*
CTS moderate	29 (37.7%)	8 (20%)	
CTS severe	17 (22.1%)	10 (25%)	
Unknown	29 (37.7%)	16 (40%)	

In patients with preoperative NCS results, CTS results were different between patients with and without bariatric surgical history as more bariatric surgery patients had mild CTS prior to CTR (10% vs. 0%; p=0.029). Bariatric surgery patients were also more likely to have mild contralateral CTS on preoperative NCS in comparison to patients with no bariatric surgery history (15% vs. 2.6%; p=0.036). However, there were no differences in terms of the presence of concomitant compression neuropathies (mild cubital tunnel syndrome (p=0.342) and presence of ulnar nerve neuropathy at the wrist (p=1.000)) based on preoperative NCS results. Additionally, there were no differences in the laterality of surgery between patients with and without a history of bariatric surgery (right: 57.9% vs. 67.9%; p=0.324) (Table 1). 

There were no major complications requiring additional surgical intervention or readmission in the cohorts evaluated. The rate of overall minor complications (p=0.298) and specific complications (p=0.779) were similar between patients with and without a history of bariatric surgery. Overall, 41 (97.6%) patients with a history of bariatric surgery had complete relief of symptoms in comparison to 73 (86.9%) patients without bariatric surgical history; however, this was not a statistically significant difference (p=0.060) (Table 2).

**Table 2:** Complications and further treatment after open CTR Data is presented as N (%). PT: physical therapy; OT: occupational therapy; CHT: certified hand therapy; CTR: carpal tunnel release

Variable	CTR only	Bariatric surgery and CTR	P-value
Minor complication	13 (15.5%)	3 (7.14%)	0.298
Minor complication type			
Infection	2 (2.38%)	0 (0%)	0.779
Persistent symptoms	1 (1.19%)	0 (0%)	
Recurrent symptoms	9 (10.7%)	3 (7.14%)	
Other	1 (1.19%)	0 (0%)	
Further treatment	10 (11.9%)	3 (7.14%)	0.541
Further treatment type			
Injection	1 (1.19%)	2 (4.76%)	0.206
PT/OT/CHT	8 (9.52%)	1 (2.38%)	
Other	1 (1.19%)	0 (0%)	
Complete relief of symptoms	73 (86.9%)	41 (97.6%)	0.060
Subsequent contralateral CTR	40 (47.6%)	16 (38.1%)	0.410

Regarding PROMs, there were no differences in both PCS-12 and MCS-12 scores at any time points evaluated. Patients with a history of bariatric surgery tended to gain a larger change in PCS-12 scores (ΔPCS-12) (6.71±11.61 vs. 3.25±12.9; p=0.165) between preoperative and one-year postoperative scores; however, this was shown to not be a statistically significant difference. Both bariatric and non-bariatric patients experienced a negative benefit in terms of change in MCS-12 (ΔMCS-12) (-0.76±9.24 vs. -0.27±9.09; p=0.795) between preoperative and one-year postoperative scores (Table 3).

**Table 3:** PROMs Data is presented as mean (standard deviation). PROMs: patient-reported outcome measures; PCS-12: SF-12 Physical Component score; MCS-12: SF-12 Mental Component score

Variable	CTR only	Bariatric surgery and CTR	P-value
Preoperative PCS-12	40.6 (9.97)	37.3 (8.44)	0.089
1-year postoperative PCS-12	43.8 (10.9)	43.4 (10.1)	0.642
Δ1-year PCS-12	3.25 (12.9)	6.71 (11.61)	0.165
Preoperative MCS-12	55.6 (8.9)	53.9 (9.97)	0.584
1-year postoperative MCS-12	55.3 (9.21)	53.6 (8.29)	0.100
Δ1-year MCS-12	-0.27 (9.09)	-0.76 (9.24)	0.795

## Discussion

In this study of 126 patients who underwent open CTR, those with or without a history of bariatric surgery experienced no differences in patient-reported outcomes at one year postoperatively. However, patients with a history of bariatric surgery tended to receive a larger benefit from surgery in terms of PCS-12 scores, but this was not statistically significant. Additionally, bariatric and non-bariatric patients had similar rates of postoperative complications and need for further treatment such as corticosteroid injections, physical therapy, occupational therapy, or hand therapy. 

Previous literature has established the association of obesity with an increased risk of developing CTS; however, there is limited and inconclusive literature regarding its association with surgical outcomes following CTR. Studies have shown obesity to be associated with increased CTS electrodiagnostic study severity and overall risk for CTS [15,16]. With regard to CTR outcomes, Allen et al. evaluated 142 patients undergoing open CTR and showed that obese patients had smaller improvement in pain scores at two and six weeks postoperatively. Additionally, obese patients underwent surgery at a younger age with an average of 46.9 years old [17]. However, a study of 493 patients undergoing open CTR showed patients with a BMI above 30 to have comparable postoperative improvement in the QuickDASH patient-reported scores to overweight and normal BMI patients [18]. While these studies evaluated CTR outcomes in obese patients, there is still no consensus on outcomes, and the association of bariatric surgery was not included in their analysis. In this study, we elucidated the association between prior bariatric surgery and open CTR postoperative outcomes. 

Interestingly, patients with a history of bariatric surgery had more mild CTS severity in comparison to the control non-bariatric patients on NCS. Neuropathy concerns have previously been established as a complication following bariatric surgery as 5-16% of those who undergo bariatric procedures may experience early peripheral nerve injury, polyradiculopathy, or even Wernicke's encephalopathy [13,19,20]. Additionally, the most common later neurologic complications include optic neuropathy, myelopathy, and peripheral neuropathy. Mechanical mechanisms have been proposed for these phenomena such as rapid weight loss leading to peripheral nerves becoming susceptible to compression due to loss of subcutaneous tissue and fat pads [21]. However, the most reported mechanism for peripheral neuropathy is nutritional deficits due to malabsorption of vitamin B12, copper, and thiamine leading to variable neuraxis dysfunction [22,23]. Studies regarding NCS results have also shown that obese patients are more likely to develop CTS and other upper extremity compression neuropathies but those obese patients who underwent bariatric surgery were significantly less likely to develop CTS or other upper extremity compression neuropathies [24]. Additionally, in a prospective study of 32 patients with a BMI >35 who underwent sleeve gastrectomy, the severity of CTS on NCS was significantly improved after surgery [25]. While our preoperative NCS study results correlate with prior evidence that CTS severity is milder in those with a history of bariatric surgery, we found that bariatric surgery history did not lead to a larger improvement in PROMs in comparison to non-bariatric CTR patients. 

As the literature regarding obesity and its association with CTR outcomes is still limited, this topic still requires further investigation. Obesity has previously been shown to cause neuropathies and is one of the most common risk factors for the development of CTS [16,26,27]. Due to advancements in weight loss surgery and management, there are now more approved surgical techniques and nonoperative treatment options to aid weight loss. Most notably, injectable semaglutides, including Ozempic and Wegovy, and other medications have become increasingly popular. A recent study by Watanabe et al. showed that the monthly growth rates of individual patients utilizing Ozempic and Wegovy for weight loss were 83.9% and 119.2%, respectively [28]. However, the literature regarding their effect on developing or worsening peripheral neuropathies is non-existent. This study and previous NCS result literature show that patients can feel comfortable to safely undergo bariatric surgery without concerns about developing more severe CTS and experiencing worse functional outcomes after CTR. 

This study is not without limitations. First, due to its retrospective nature, there is potential for inherent selection bias of the patients included for analysis. Second, patients with a history of bariatric surgery were identified through manual chart review. It is possible that more patients may have fit the inclusion criteria of this study but had missing information and no documentation of bariatric surgical history in the EMR. Third, while bariatric surgical history was documented in the EMR, the interval between bariatric surgery and CTR was not documented in a significant portion of the patients included in this study (40%). Therefore, we could not accurately evaluate the association of interval between surgeries and CTR surgical outcomes. Fourth, the mean BMI prior to and one year after bariatric surgery have been previously reported as 48 and 33, respectively [29]. However, we were unable to report the mean BMI prior to bariatric surgery and the amount of weight loss experienced postoperatively in our patient cohort due to limitations of EMR documentation. Fifth, only 42 patients with a history of bariatric surgery were included in the final cohort, and we were only able to include outcomes at one year postoperatively. Therefore, this study may have been underpowered to establish surgical outcome differences. Future research should be conducted using larger patient cohorts with confirmed bariatric surgery history as well as assess outcomes over longer follow-up periods. Sixth, while we performed a propensity match with control patients based on age, sex, race, BMI, CCI, smoking status, and history of diabetes mellitus, it is possible that other confounding variables may have affected the results of this study. However, we feel that the propensity match allowed for minimal confounders as we utilized previously documented patient risk factors associated with differences in CTR outcomes in the match. Finally, the PROMs utilized in this study were limited to those available at our institution. More specific outcome measures for CTR such as the Boston Carpal Tunnel Questionnaire (BCTQ), Michigan Hand Outcomes Questionnaire (MHQ), or Disabilities of the Arm, Shoulder, and Hand (DASH) may have further elucidated differences in postoperative outcomes [30].

## Conclusions

This study explored the association between preoperative history of bariatric surgery and surgical outcomes following open CTR. Patients with a history of bariatric surgery tended to have a larger benefit from open CTR in comparison to the control cohort; however, this was not a significant difference. The current literature regarding CTR outcomes as it relates to obesity and bariatric surgery is quite limited; therefore, the association should be explored further. Future research should continue to investigate the association of bariatric surgery with postoperative surgical outcomes following open CTR with larger patient cohorts or through multi-center collaboration. 
